# IFN-γ differential expression in the hypothalamus-pituitary-ovary axis of thyroidectomized rats

**DOI:** 10.1186/s12902-022-01223-z

**Published:** 2022-12-14

**Authors:** Jingjie Wei, Yan Liang, Ningbo Jiang, Ge Hu

**Affiliations:** 1 Animal Science and Technology College, Beijing University of Agriculture, No.7 Beinong Road, Changping, Beijing, 102206 China; 2 Beijing Key Laboratory of TCVM, Beijing University of Agriculture, No.7 Beinong Road, Changping, Beijing, 102206 China

**Keywords:** Hypothyroidism, IFN-γ, Ovarian, Hypothalamus–pituitary–thyroid axis, Rat

## Abstract

IFN-γ is critical for both thyroid and ovarian function, while thyroxine, secreted from the thyroid gland, regulates the ovarian function via the hypothalamus-pituitary -ovary axis. However, the effect of thyroxine on INF-γ involved in the regulation of hypothalamic pituitary ovarian axis ovarian function is hitherto unknown. Therefore, we set up three groups including a sham-operated group, an experimental thyroidectomized group, and an experimental thyroidectomized group treated with T4 to reveal the IFN-γ expression levels in the in the hypothalamus, pituitary gland, and ovary by immunohistochemical staining, RT-PCR, and Western blotting. IFN-γ-like immunoreactive-positive substances were visualized in the hypothalamus, pituitary gland, and ovary, which were located mainly in the cytoplasm of the hypothalamic neurons anterior pituitary cells, luteal cells, and theca cells in the ovary of hypothyroidism rats, respectively. RT-PCR and Western blotting showed that the rats in the experimental thyroidectomized group treated with T4 had significantly elevated expression of IFN-γ at both the mRNA and protein levels. Thyroxine affects the expression of IFN-γ in the thalamus-pituitary-ovarian axis, which may influence the secretion of IFN-γ to regulate ovarian function during hypothyroidism. This work highlights the potential effect of thyroxine on the involvement of INF-γ in the modulation of the ovarian function in the hypothalamic-pituitary-ovarian axis.

**Supplementary Information:**

The online version contains supplementary material available at 10.1186/s12902-022-01223-z.

## Introduction

Accumulating evidence has confirmed that the hypothalamic-pituitary-thyroid axis plays a key role in female reproduction in humans and animals [1, 2]. The function of the thyroid gland, which is involved in the hypothalamic-pituitary-thyroid axis, is critical for the development and function of cardiovascular, nervous, immune, and reproductive systems [3–5]. Thyroxine participates in the metabolism of various substances through the hypothalamic-pituitary-ovarian axis and the regulation of the development of the gonads and the maintenance of normal female reproductive and endocrine functions [6]. In addition, thyroxine also regulates ovarian function and maintains the balance of reproductive endocrine by affecting the secretion of pituitary gonadotropin through the hypothalamus-pituitary-ovary axis [7, 8]. Altogether, thyroxine is involved in almost all stages of female reproductive physiological activities.

Recent research revealed that the altered levels of,3’-,5,5’tetra-iodothyronine (T4) and 3,3’,5-triiodothyronine (T3), the major circulating forms of thyroid hormones, can influence mammalian fertility [9]. Human ovarian granulosa cells, stromal cells and oocytes all expressed thyroxine receptor (TR) and thyroid-stimulating hormone receptor (TSHR) [10, 11], suggesting that the ovary is also one of the target organs of thyroxines and thyroid-stimulating hormone (TSH). In the ovary, thyroid hormones (THs), namely triiodothyronine (T3) and thyroxine (T4) act as modulators of several physiological processes such as steroidogenesis, oocyte formation and maturation of granulosa cells, follicular development and differentiation, and ovulation. T4 is the precursor of the active thyroid hormone T3, which further binds to the thyroid receptors (TRs) [9–12]. Moreover, thyroid dysfunctions have been associated with disturbed folliculogenesis, impaired ovulation and fertilization rate, and, in severe cases, complete ovarian failure or cancer [13–15].

Reportedly, the hypothalamus-pituitary-thyroid (HPT) axis is involved in many diseases or acute stress with elevated or reduced thyroid hormone levels, affecting the immune system in a variant fashion [16, 17]. Studies on thyroid gland pathogenesis of and other autoimmune diseases showed that some cytokines such as interleukin-1 (IL-1) and interferon-gamma (IFN-γ) have close correlation with hypothyroidism, the immune regulation induced by which maintains the autoimmune response and directly influences thyroid gland function [18]. Increased IFN-γ in turn stimulates Th1 chemokines release from thyrocytes, initiating and perpetuating the autoimmune process in patients with hyperthyroidism [19]. Therefore, IFN-γ may be a humoral mediator in the pathogenesis of hypothyroidism in vivo. In addition, IFN-γ seems to play an important role in the tissue repair of the mammalian reproductive system. IFN-γ regulates ovarian function in mammals through participating in the degeneration of the corpus luteum. It also indirectly affects the production of steroids and follicular development by inhibiting follicle-stimulating hormone (FSH)-induced IL-6 [20].

A regulatory relationship between thyroxine and hypothalamus-pituitary-ovary axis has been observed in earlier clinical research, but the definite mechanism has not been established. In a previous study, the levels of TNF-α, IL-6, and other cytokines increased in hypothyroidism rats [21]. Thus, we speculated that hypothyroidism had an impact on the growth, development, and differentiation of cells in ovarian tissues via altering the expression of IFN-γ in the hypothalamus-pituitary-ovarian-axis. We also aimed to elucidate whether IFN-γ had a direct link during this process. hypothalamus-pituitary-ovary axis. Therefore, to explore the role of thyroxine in the hypothalamus-pituitary-ovary axis and its relationship with IFN-γ, we detected the expression of IFN-γ in the hypothalamic-pituitary-ovarian axis of hypothyroidism rats at the mRNA and protein levels. The result will pave the path for research on hypothyroidism that influence the function of the reproductive system at the ovarian level through the immune system.

## Materials and methods

### Ethics statement

Sprague Dawley rats were purchased from the animal center of the Genetics Institute, Chinese Academy of Sciences, Beijing, China (Certificate Number: SCXK-PLA 2012–0004). All animal experiments were conducted after an approval was obtained from the Institutional Animal Care and Use Committee at the Academy of Military Medical Sciences Institute (Beijing, China; approval no. SYXK2014-0002).

### Animal treatments

**Fig. 1:**
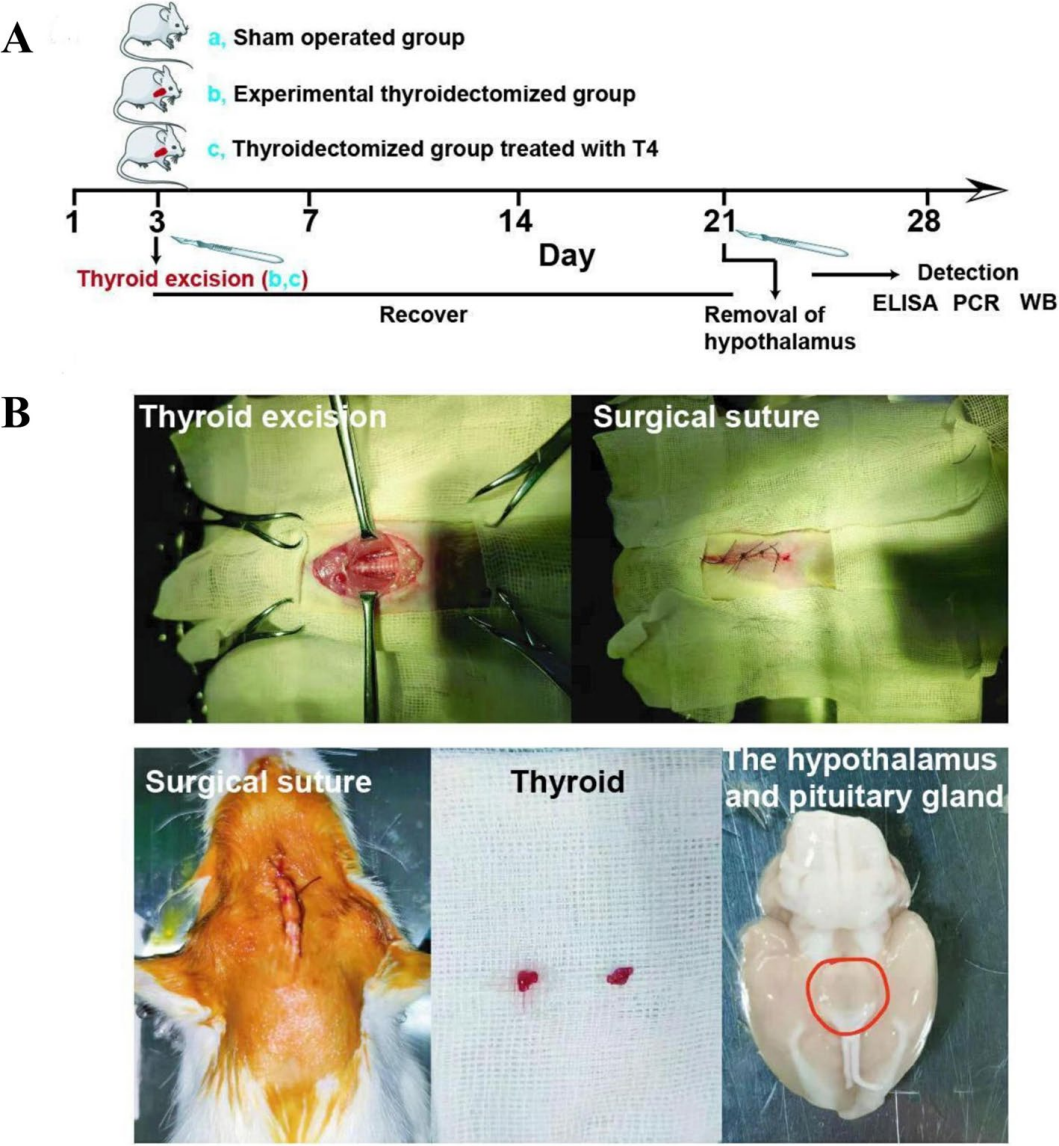
The levels of serumT3 and T4 were reduced in the thyroidectomized rats, but the levels of serum TSH followed the opposite trend. **A** The experimental workflow: **B** Surgical picture of the thyroid excision

Eighteen female rats (200 ± 20 g) were randomly divided into three groups. In the sham-operated group, thyroid glands of the rats were exposed but not removed after anesthesia. After stitching, the rats continued to be reared. In the experimental thyroidectomized group, the rats were anesthetized by intraperitoneal injection of 1% sodium pentobarbital (30 mg/kg) [22], and bound on the operating table in the supine position. Approximately 5 cm of the skin at the midline of the ventral side of the neck was incised. Then, the larynx and trachea were exposed, and the thyroid gland on the bilateral sides of the trachea behind the throat was removed (Fig. 1). In the thyroidectomized group treated with T4, the rats were fed normally after removing the thyroid gland following the aforementioned method, and injected with thyroxine (T1775, Sigma, St Louis, MO, USA) intramuscularly, 0.02 mL/per time, seven consecutive days. After 14 days’ normal feeding, the rats in the three groups were anesthetized by intramuscular injection of 1% pentobarbital sodium (30 mg/kg). Blood samples were collected from the heart and centrifuged (3000 g). The serum was stored at 2 °C–8 °C for radioimmunoassay.

### Tissue separation

**Fig. 2:**
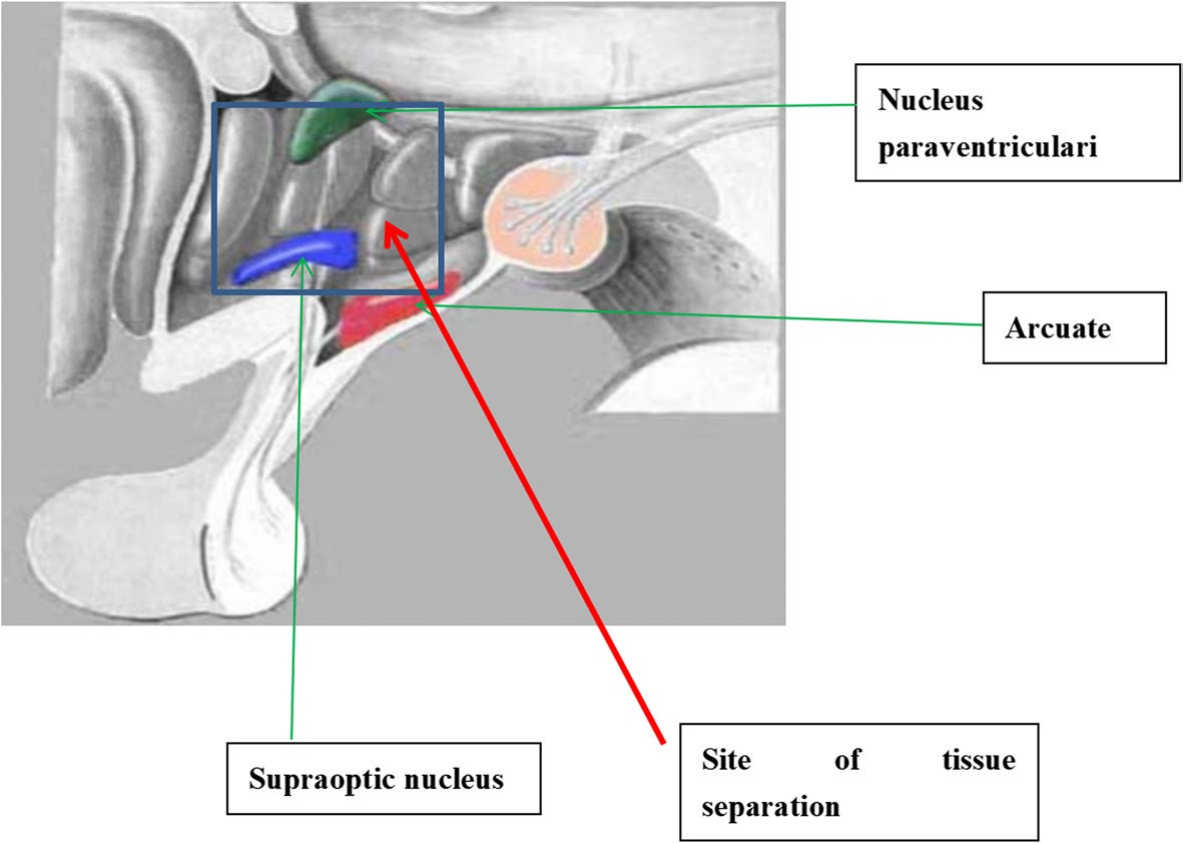
The hypothalamus was located at the ventral side of the diencephalon, the hypothalamus, the dorsal optic tract, and the base of the third ventricle of the supraoptic and paraventricular nuclei. The hypothalamus tissues were separated by surgery

All rats were substituted with cervical dislocation, the hypothalamus, pituitary, and ovary tissues were surgically separated (Fig. 2). The tissues were placed in precooled saline and immediately prepared for the following experiments. All the tissues were separated into three parts, which were then differently prepared as follows. One part was fixed with 4% paraformaldehyde (pH 7.4) at 4 °C for 24 h and then embedded in paraffin for immunohistochemical analysis. Another part of the hypothalamus, pituitary gland and ovary tissues were immediately frozen in liquid nitrogen and stored at—80 °C for RT-PCR assay. The remaining tissues were subjected to Western blotting.

### Radioimmunoassay

Detection of T3/T4 was carried out following the manufacturer's instruction of the iodine [125I] thyroxine radioimmunoassay kit (approval No.: guoyaozhunzi s10930044). Briefly, 100 μL of mouse standard and spare serum sample was transferred into a labeled test tube (NSB tube plus 200 μL of Zero standard). Except for the NSB tubes, 100 μL thyroxine antiserum was added into all tubes, which were then incubated at 4 °C for 24 h. Next, 100 μL of 125I labeled thyroxine antigen was added into each tube, and the samples were incubated at 37 °C for 45 min. Further, 500 μL of separating agent was added into each tube and mixed well, followed by incubation at room temperature for 15 min. After centrifugation at 3500 g for 15 min, the supernatant was immediately aspirated, and the volume flow of the sediment in each tube was determined by a FJ-2008 γ counter. The content of T3/T4 in each tube was calculated according to the standard curve and the B/BO percentage of the sample tube (B: sedimentation count of each tube—precipitation count of the non-specific tube; BO: precipitation count of the zero-standard tube—precipitation count of the non-specific tube). Both the experimental thyroidectomized group treated with T4 and the sham-operated group were determined in the same batch.

### Immunohistochemistry

The procedure for IFN-γ immunohistochemical detection was similar to that described in a previous report [23]. The hypothalamus, pituitary gland, and ovary tissues were embedded in paraffin; pituitary gland and ovary tissues of serial 5-μM sections were obtained. Then, the sections were deparaffinized in xylene and rehydrated in graded ethanol. Antigen retrieval was performed by microwaving the sections for 16 min (four times for 4 min) at full power in 0.01 M sodium citrate buffer (pH 6.0). The tissue sections were then treated with 3% H_2_O_2_; non-specific binding was blocked with 10% normal donkey serum. rat IFN-γ antibody (I5027, 1:100, Sigma-Aldrich, St Louis, MO, USA) was subsequently added, followed by incubation at 4 °C for 24 h. After further washing in PBS, the sections were incubated with rabbit anti-rat IgG (1:50; GBI, Washington state, USA) for 2 h at room temperature. After the sections were rinsed with PBS, horseradish enzyme-labeled Streptomyces ovalbumin working solution (GBI, Washington state, USA) was added for 2 h, and the peroxidase activity was detected with diaminobenzidine. Finally, the sections were counterstained with hematoxylin and mounted using conventional methods.

### RT-PCR

The hypothalamus, pituitary gland, and ovary tissues were collected as specified above and grinded with liquid nitrogen. Total RNA was isolated using Trizol reagent (Invitrogen; Carlsbad, USA) and dissolved in 50 μL RNasefree water. We added 1 mL of Trizol to every 50–100 mg powder, shacked well, and placed the samples at 4 °C for 15 min. Then, chloroform was added in a ratio 0.2 mL chloroform/ 1 mL Trizol. The samples were mixed well by vortex oscillation for 30 s, placed at 4 °C for 2–3 min, and centrifuged at 12,000 g for 15 min at 4 °C. After centrifugation, the supernatant was transferred to a new tube, followed by the addition of isopropanol in a ratio 0.5 mL isopropanol/ 1 mL Trizol, and the samples were evenly mixed. Next, the supernatant was placed at—20 °C for 10 min, and centrifuged at 12,000 g for 15 min at 4 °C. Isopropanol was discarded, and 1 mL of 75% ethanol was added. Further, the precipitate block was flipped up with fingers, inverted several times, and centrifuged at 7500 g for 5 min at 4 °C. 75% ethanol was discarded and the precipitate block was dried naturally in ultra-clean table for 15–30 min. Nuclease-free water was added into the precipitate block to promote dissolution at 65 °C for 5–10 min. The amount of RNA was determined by UV spectrophotometer, and the integrity of RNA was detected by agarose gel electrophoresis. Reverse transcription was performed using a one-step RT-PCR kit (Takara; Tokyo, Japan). The primers that were used are displayed in Table 1. The fold changes in the gene expression were calculated using the 2 − ^ΔΔCt^ method [24]. Quantification of related mRNA level was normalized to *GAPDH* mRNA level.

**Table 1:** Sequences used for quantitative RT-PCR

Gene	Sequences (5’ to 3')
*Ifng-F*	GAGGAACTGGCAAAAGGACG
*Ifng-R*	CAGGTGCGATTCGATGACAC
*GAPDH-F*	TGCTGAGTATGTCGTGGAG
*GAPDH-R*	GTCTTCTGAGTGGCAGTGAT

### Western blotting

After weighing the hypothalamus, pituitary gland and ovary, RIPA lysate was added at the ratio of weight: lysate volume equal to 1:9. The tissue homogenate was centrifuged three times at 15,000 g for 10 s. After incubation on ice for 20 min, the supernatant was centrifuged at 4 °C and 13,000 g. Then, the supernatant was transferred to PVDF membrane by SDS-PAGE (Sigma), which was subsequently taken out and soaked in 3% BSA-TBST, and then sealed for 30 min by shaking at room temperature. After the blocking solution was discarded, IFN-γ antibody was added at a dilution ratio 1:10,000, and the solution was incubated for 10 min at room temperature and then overnight at 4 °C. The membrane was washed five times with TBST for 3 min each time, and incubated 40 min at room temperature with the secondary anti mouse IgG (H + L) HRP at the dilution ratio of 1:10,000. Then wash the membrane by TBST 6 times, for 3 min each time. After ECL was added to the PVDF membrane for 3–5 min, the membrane was exposed for 10 s to 5 min (the exposure time was inversely proportional to the light intensity). Finally, developed for 2 min, and performed fixation. The gray values of protein bands were quantified by ImageJ software. Quantification of related proteins was normalized to β-actin levels.

### Statistical analysis

Significant differences and *P*‐values were determined using unpaired *t*‐test (two groups) or one‐way ANOVA (multiple groups) with GraphPad Prism 9 software. All data are expressed as means ± SD (n = 6).

## Results

### The levels of serum T3 and T4 in thyroidectomized rats were lower

**Fig. 3:**
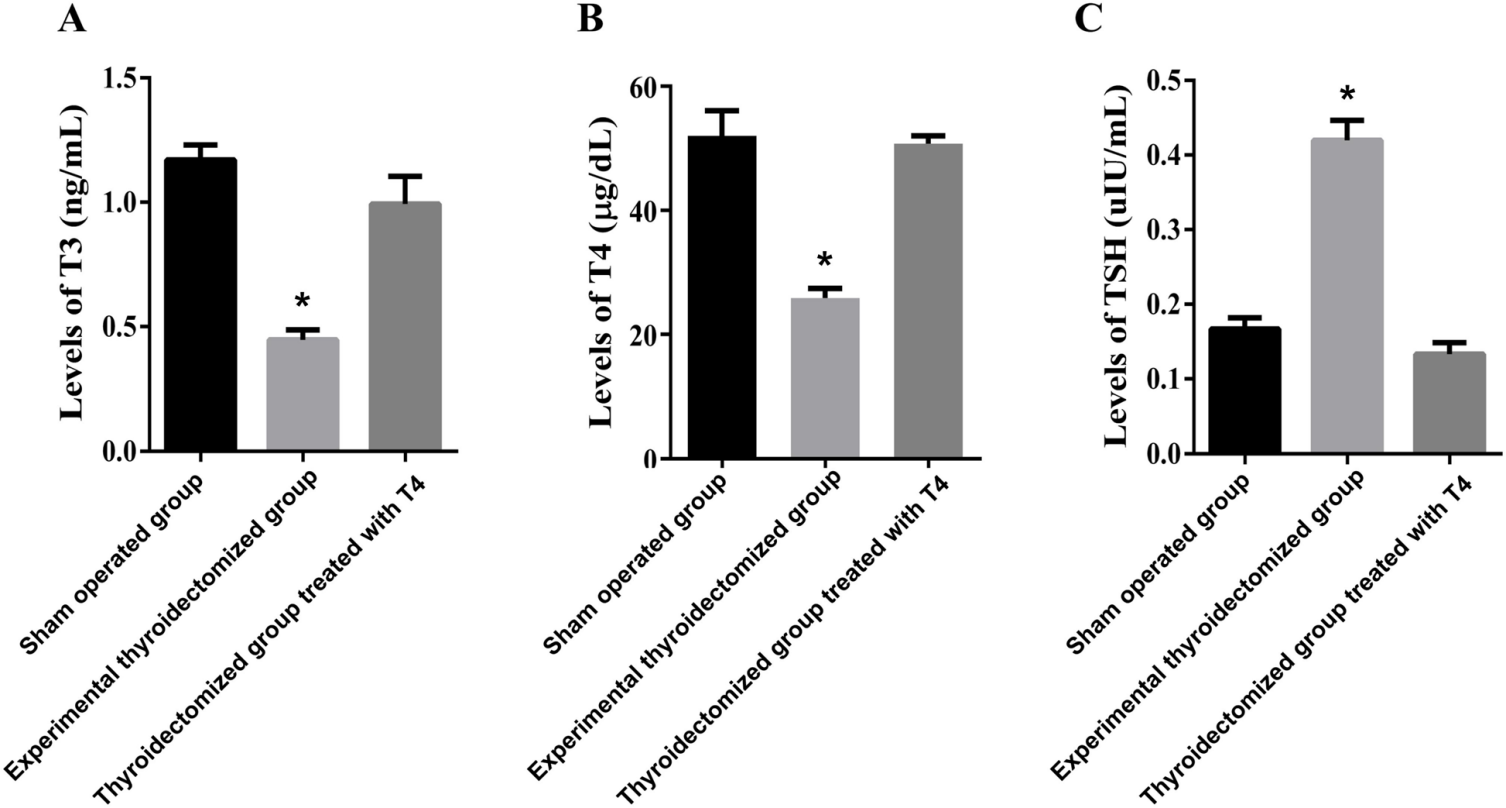
The levels of serum T3 and T4 were decreased in the thyroidectomized rats. **A** The levels of serum T3 in the rats; **B** The levels of serum T4 in the rats; **C** The levels of serum TSH in the rats.^*^P < 0.05

To confirm that thyroidectomization affected the thyroid hormones, the serum TSH, T3, and T4 levels were directly detected by RIA in the three groups, including the sham-operated thyroidectomized, the experimental thyroidectomized, and thyroidectomized treated with T4 groups (Fig. 3). Our results showed that the levels of T3 and T4 were significantly lower in the experimental thyroidectomized group *(P* = 0.035, *P* = 0.033), while the TSH were higher *(P* = 0.029). In addition, the decreased T3 and T4 levels in the thyroidectomized group with the addition of T4 recovered but no significant effect was exerted on the TSH levels. These results suggested that T4 might restore the function of thyroid hormones (T3/T4) in the thyroidectomized rats.

### IFN-γ was detected in the hypothalamus, pituitary gland, and ovaries of thyroidectomized rats

**Fig. 4:**
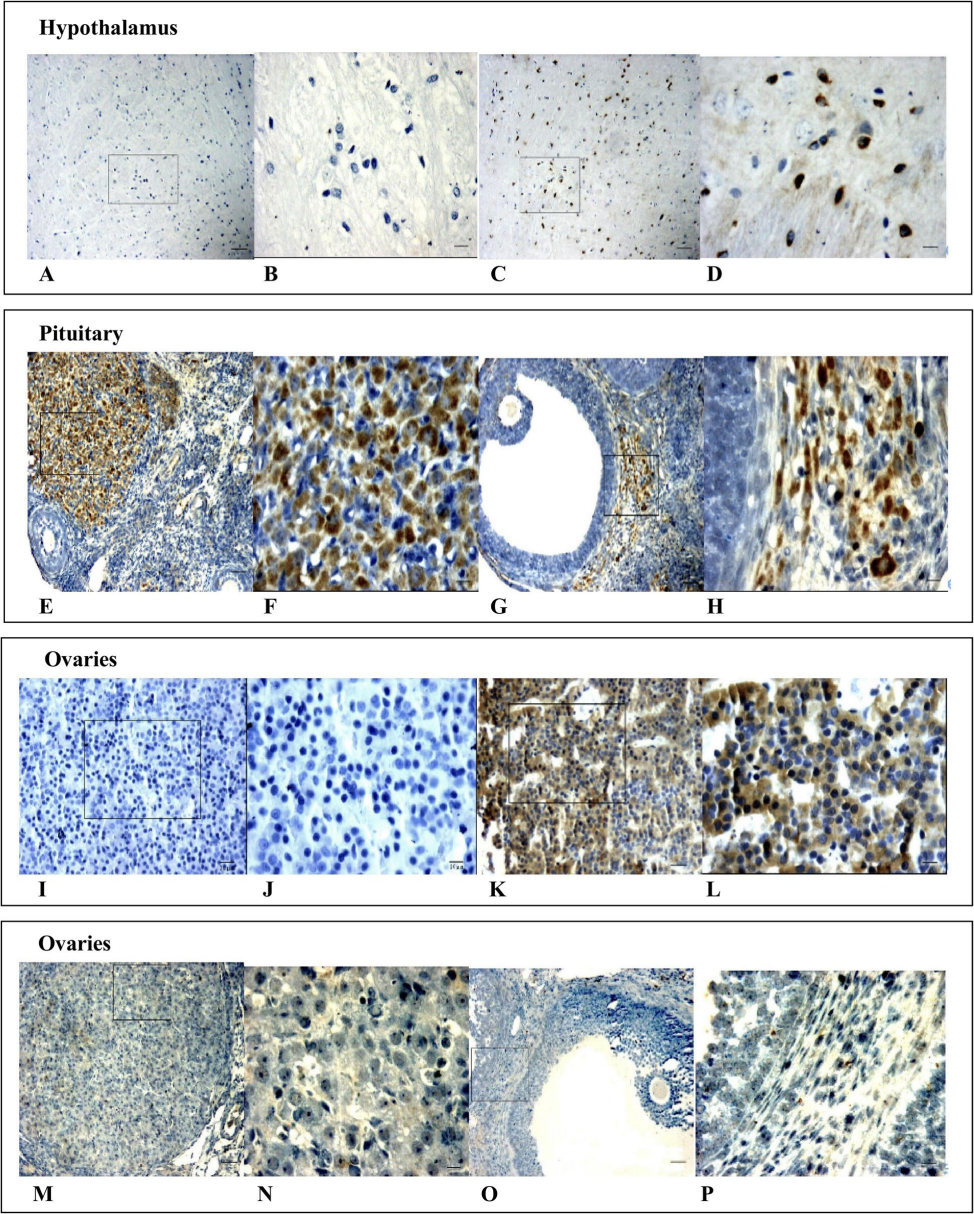
IFN-γ was localized in the hypothalamus, pituitary gland, and ovary of the thyroidectomy rats. Rat IFN-γ of in the hypothalamus, pituitary gland, and the luteum, and IFN-γ in the follicles are presented in the images. **A** and **B** The hypothalamus of the experimental thyroidectomized group treated with T4; **C** and **D** The hypothalamus of the experimental thyroidectomized group; **E** and **F** The pituitary gland of the sham-operated group; **G** and **H** The pituitary gland of the experimental thyroidectomized group; **I**–**L** The corpus luteum and the follicles of the experimental thyroidectomized group with T4 addition; (M–P) The corpus luteum and the follicles of the experimental thyroidectomized group. The scale bar in **A**, **C**, **E**, **F**, **G**, **I**, **K**, **M**, and **O** indicates 50 μm, whereas that in **B**, **D**, **F**, **H**, **J**, **L**, **N**, and **P** represents 10 μm

To further investigate the functional role of IFN-γ in the thyroidectomized rats, immunohistochemistry assay was employed to determine the expression and location of IFN-γ in the tissues. No positive staining of IFN-γ was observed in the hypothalamus, pituitary glands, and ovary of rats in the treatment or sham-operated groups. IFN-γ immunoreactive-positive substances were detected in the hypothalamus, pituitary gland, and ovary of rats of the experimental thyroidectomized group treated with T4 (Fig. 4). In the hypothalamus, they were located in the cytoplasm of neurons with around 50% of them with positive staining observed by a light microscope. In the pituitary gland, they were situated in the cytoplasm of the pars distalis adenohypophysis, and arranged closely IFN-γ-positive cells constituted approximately 90%. No IFN-γ immunoreactive-positive substance was found in the neurohypophysis or other parts of the pituitary gland. In the ovary, IFN-γ immunoreactive-positive substances were mainly detected in the cytoplasm of luteal and thecal cells, about 60% of which were positive. In addition, no positive substances were observed in other parts of the ovary.

### Increased IFN-γ in the hypothalamus, pituitary gland, and ovary tissues

**Fig. 5:**
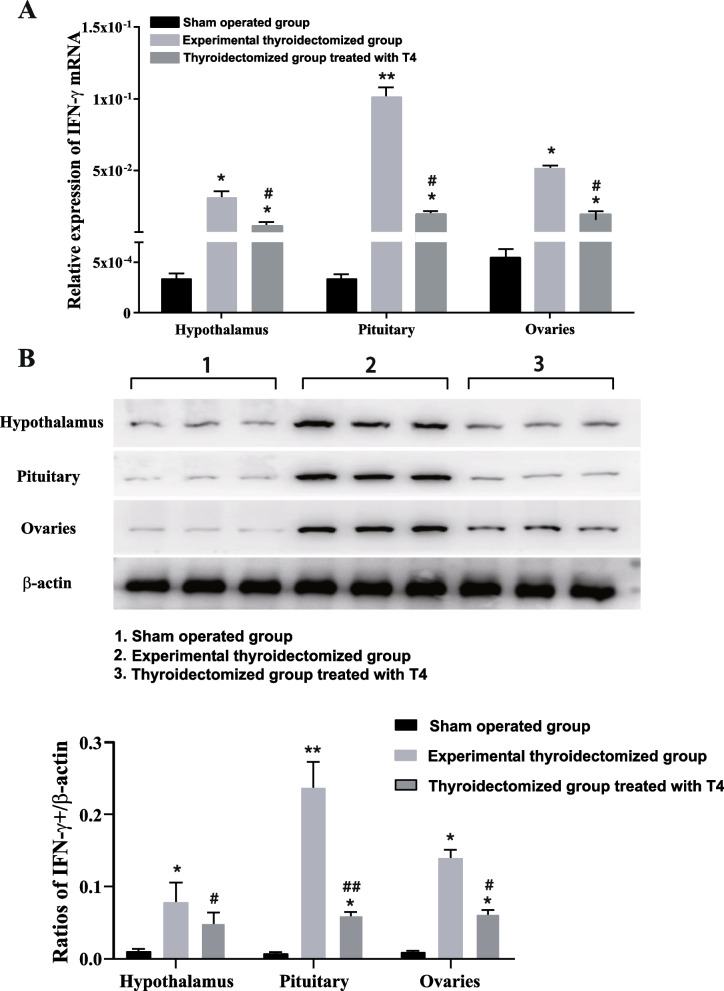
mRNA and protein levels of IFN-γ. **A** The relative expression of IFN-γ was detected by RT-PCR assay. The values representing the mRNA level were relative to the internal reference gene *GAPDH*;^*^P < 0.05, ^**^P < 0.01, ^#^*P* < 0.05 **B** The protein levels of IFN-γ in the hypothalamuses and the pituitary gland and ovary glands of each experimental group. Relative expression of IFN-γ to β-actin. ^*^P < 0.05, ^**^P < 0.01, ^#^*P* < 0.05, ^##^*P* < 0.01. Full-length blots are presented in Supplementary Figures S1

To quantify the mRNA level of IFN-γ in the hypothalamus, pituitary gland and ovary tissues (Fig. 5A), RT-PCR results showed that the experimentally thyroidectomized group was significantly higher than that of the sham-operated group (*P* = 0.017, *P* = 0.009, *P* = 0.021), while the treatment group was found to have significant differences of IFN-γ mRNA level only in pituitary gland and ovary from that of sham-operated group (*P* = 0.040, *P* = 0.041), and significantly decreased mRNA expression of IFN-γ compared with experimental thyroidectomized group (*P* = 0.012, *P* = 0.032). The expression of IFN-γ in the hypothalamus of the treatment group was significantly higher than that in the sham-operated group (*P* = 0.037), but lower than that in the experimental thyroidectomized group (*P* = 0.043).

### Thyroidectomy significantly increased IFN-γ protein levels in the hypothalamus, pituitary gland, and ovary

Western blotting was used for detect determine the IFN-γ levels in the hypothalamus, pituitary gland, and ovary tissues, of the sham-operated thyroidectomized, the experimental thyroidectomized group, and the thyroidectomized treated with T4 rat groups (Fig. 5B). Data showed that the expression of the IFN-γ protein in the pituitary gland and ovary of the treatment group was higher than that of the sham-operated group (*P* = 0.045, *P* = 0.042)), but was significantly lower than that of the experimental thyroidectomized group (*P* = 0.038, *P* = 0.008, *P* = 0.026). Altogether, IFN-γ was increased both in mRNA and protein to respond the thyroidectomized rats and T4 supplement inhibited the increased IFN-γ, suggesting that IFN-γ can serve as the indicator in the participate the thyroxine modulate the ovarian function in the hypothalamic-pituitary-ovarian axis.

## Discussion

As a metabolic hormone, TH maintains the body's function via multiple target organs, and its abnormal secretion may induce diverse endocrine diseases, influencing the structure and function of many organs and systems, including the reproductive system [25]. In recent years, increasingly more research has been conducted to investigate the impact of thyroid disease on reproductive function [26–28]. A large number of experimental studies have shown that thyroid dysfunction affects reproductive hormone levels in female animals [7]. Previous reports have revealed that the ovaries are vulnerable to immune attack, leading to diseases such as premature aging and polycystic ovary. Taken together, hypothyroidism is related to ovarian disease since thyroxine tablet treatments mitigate the clinical symptoms and promote functional restoration of the ovary in patients with both disorders. However, it remains elusive whether hypothyroidism has a profound effect on the growth, development, and differentiation of ovarian cells by changing cytokine levels. Moreover, thyroid disease is also an autoimmune disease [29, 30], suggesting a close link between them.

IFN-γ is produced by a variety of cells and possesses diverse biological activities, such as antiviral, antitumor, and immune regulation [31]. In addition, it participates in regulating reproductive, endocrine, metabolic, and other physiological activities [32, 33]. Generally, previous reports on the association between cytokines and thyroid function have been focused on TNF-α, IL-1, and IFN-γ, all exerting an inhibitory effect on thyroid activities. However, conflicting results have been obtained on the role of IFN-γ in the pathological process of adult hypothyroidism induced by autoimmune thyroiditis. In this study, a hypothyroidism model was established by surgical removal of the thyroid gland. In addition, based on SP and H_2_O_2_-DAB yellow coloring method, RT-PCR, and Western blotting, IFN-γ was detected in the hypothalamus, pituitary gland and ovary. The results obtained here suggest that interferon-γ may play a landmark role in the abnormalities of the hypothalamus-pituitary-ovary axis and reproductive function caused by hypothyroidism. Thus, it can serve as a new index for the detection of the impact of hypothyroidism on the reproductive system function through cytokines at the ovarian level.

Cytokines are associated with ovarian function, but the interaction between the immune and reproductive systems seems to be rather complicated. Studies on mammals showed that immune cells participate in the regulation of the hypothalamus-pituitary-ovary axis. Interestingly, cytokines are involved in every link of the thalamus-pituitary-ovary axis, and even affect information transmission, by connecting different systems (the immune, nervous, endocrine, and endothelial systems). Ovary is considered to be the main interaction organ between the immune and the endocrine systems [34, 35]. In addition, IFN-γ is also crucial for tissue repair of mammalian reproductive system [36], which is mediated by related immune responses. IL-1B, IL-6, IFN-γ, and TNF-a and other cytokines were found to act as key modulators in this regard [37, 38]. Therefore, IFN-γ may play a critical role to trigger the innate immunity in the ovary in the hypothalamus-pituitary-ovary axis of rats with hypothyroidism.

A previous study showed that hypothyroidism rats had increased levels of TNF-α, IL-6, and other cytokines [39]. Therefore, we hypothesized that hypothyroidism might affect the growth, development, and differentiation of the cells in the ovarian tissue by altering the cytokine levels in vivo. In this study, we preliminarily determined the effect of thyroid hormone on IFN-γ expression in the hypothalamus-pituitary-ovary axis. Our findings provide novel insights into the effects of hypothyroidism on the reproductive system function through the hypothalamus-pituitary-ovary axis at the ovarian level via the immune system. Based on our results, in our further research, we intend to detect the expression of thyroid hormone receptor protein in tissues and explore the mechanisms by which thyroid hormone affects the cytokine level in the hypothalamus-pituitary-ovary axis. Moreover, we will study the relevant signaling pathways through transcriptome analysis to explore the mechanism of thyroid hormone on the hypothalamus, pituitary gland, and ovary.

## Supplementary Information

**Additional file 1.**

## Data Availability

The datasets generated or analyzed during this study are available from the corresponding author on reasonable request.
