# Supplementary material for: IFN-γ differential expression in the hypothalamus-pituitary-ovary axis of thyroidectomized rats
Source: BMC Endocr Disord. 2022 Dec 14;22:317. doi: 10.1186/s12902-022-01223-z (PMC9749148; doi:10.1186/s12902-022-01223-z)
Supplement: Supplementary file 1 — Additional file 1. [file 12902_2022_1223_MOESM1_ESM.docx]

**IFN-γ differential expression in the hypothalamus-pituitary-ovary axis of thyroidectomized rats**

Jingjie Wei^a^, Yan Liang^b^ , Ningbo Jiang^b^ and Ge Hu ^a,*^


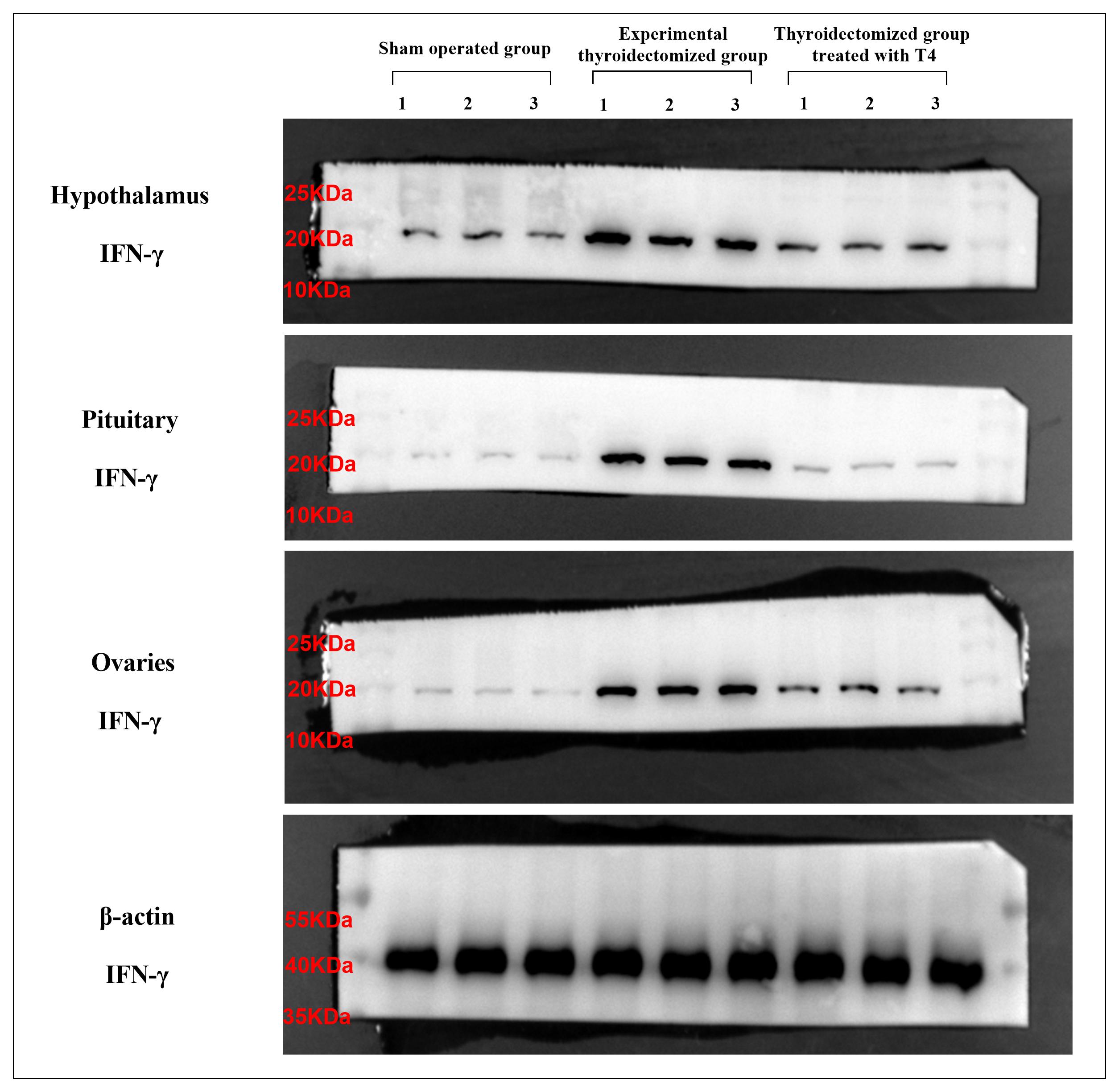


**Figure S1.** Figure S1 is an image of repeated imprinting of Figure 5 with the same sample and antibody.
